# *NOD2* Polymorphisms Associated with Cancer Risk: A Meta-Analysis

**DOI:** 10.1371/journal.pone.0089340

**Published:** 2014-02-20

**Authors:** Jingwei Liu, Caiyun He, Qian Xu, Chengzhong Xing, Yuan Yuan

**Affiliations:** 1 Tumor Etiology and Screening Department of Cancer Institute and General Surgery, the First Affiliated Hospital of China Medical University, Shenyang, China; 2 Key Laboratory of Cancer Etiology and Prevention, China Medical University, Liaoning Provincial Education Department, Shenyang, China; University of North Carolina School of Medicine, United States of America

## Abstract

**Background:**

Emerging evidence indicated that common polymorphisms of *NOD2* might impact individual susceptibility to cancer. However, the results from published studies were inconclusive. The aim of this meta-analysis was to elucidate whether *NOD2* polymorphisms were associated with cancer risk.

**Methods:**

A systematically literature search was performed by using electronic databases including PubMed and Web of Science. ORs and their 95% CI were used to assess the strength of association between *NOD2* gene polymorphisms and cancer risks.

**Results:**

Thirty case-control studies were included in this meta-analysis. The pooled analysis indicated that *NOD2* rs2066842 C/T polymorphism was not significantly associated with cancer risk; for *NOD2* rs2066844 C/T polymorphism, (TT+CT) genotype was associated with increased cancer risk compared with wild-type CC genotype (OR = 1.32, 95% CI = 1.01–1.72, P = 0.041); for *NOD2* rs2066845 C/G polymorphism, individuals with (CC+CG) genotype were significantly associated with increased cancer risk compared with GG genotype (OR = 1.32, 95% CI = 1.01–1.72, P = 0.040); for *NOD2* rs2066847 (3020insC) polymorphism, carriers of (insC/insC+insC/−) genotype were significantly associated with increased cancer risk compared with −/− carriers (OR = 1.23, 95% CI = 1.10–1.38, P<0.001). In the subgroup analysis of cancer type, (insC/insC+insC/−) genotype was significantly associated with increased risk of colorectal cancer, gastric cancer and MALT lymphoma, breast cancer, lung cancer, laryngeal cancer but not with urogenital cancer, pancreatic cancer, melanoma or non-Hodgkin lymphoma.

**Conclusion:**

*NOD2* rs2066844 C/T, rs2066845 C/G and rs2066847 (3020insC) polymorphisms might be associated with increased cancer risk. No significant association was observed between *NOD2* rs2066842 C/T polymorphism and cancer risk. Further large-scale and well-designed studies are still needed to confirm the results of our meta-analysis.

## Introduction

Cancer is a major health problem in the most parts of the world. Approximately 12.7 million cancer cases and 7.6 million cancer deaths are estimated to occur each year worldwide [1]. The prevention and treatment for cancers caused increasing financial burdens around the world [2]. As a complex disease, cancer is strongly influenced by environmental and genetic factors, of which gene polymorphism is a critical cause for the difference of individual genetic susceptibility to cancer [3]. Identification of the key gene polymorphisms that are associated with cancer risk is essential for predicting individual at risk.

The nucleotide-binding oligomerization domain containing 2 (*NOD2*) gene, also known as *CARD15*, is mapped to chromosome 16q21. NOD2 is a member of evolutionarily conserved Nod-like receptors (NLRs) family which share a tripartite structure of a C-terminal sensor domain (leucine-rich repeats, LRRs), a central nucleotide-binding oligomerization domain (NOD) and an N-terminal effector domain (CARD) [4]. NOD2 participates in sensing components of microbial cell wall and has been reported to regulate apoptosis and chronic inflammatory conditions [5]. The most commonly studied polymorphisms included three missense mutations (rs2066842 C/T, rs2066844 C/T, rs2066845 C/G) and a frameshift mutation (rs2066847 insC). These four polymorphisms were located at coding regions and might affect the expression and function of NOD2 by altering amino acid. Recently, increasing studies investigated the relations between these four polymorphisms and disease risk.

The rs2066842, rs2066844, rs2066845 and rs2066847 polymorphisms were initially found to be associated with increased risk of Crohn’s disease (CD) in 2001 to 2003 [6]. Subsequently, the relation between these polymorphisms and ulcerative colitis (UC) risk was revealed [7]. In 2004, Kurzawski et al. first linked *NOD2* polymorphism to risk of colorectal cancer [8]. After that, increasing studies focused on the association between *NOD2* polymorphisms and risks of various cancers including gastric cancer, colorectal cancer, endometrial cancer, breast cancer, ovarian cancer, laryngeal cancer and so on. However, the results from the individual studies were inconsistent.

So far, no comprehensive meta-analysis has investigated the overall cancer risk in relation to *NOD2* polymorphisms, except for a meta-analysis only concerning colorectal cancer in 2010. To explore whether *NOD2* polymorphisms were associated with risks of overall cancer and specific cancer subtypes, we performed a meta-analysis on the association between the four most frequently studied *NOD2* polymorphisms (rs2066842 C/T, rs2066844 C/T, rs2066845 C/G and rs2066847 insC) and cancer risk in the present study.

## Materials and Methods

### Identification and Eligibility of Relevant Studies

Literatures of electronic databases including PubMed and Web of Science were systematically searched using the search terms of “*NOD2/CARD15*”, “polymorphism/mutation/variant” and “cancer/malignancy/neoplasm”. References cited in each identified literatures were further searched manually to find potential available studies. We contacted the author for specific raw data if the data presented in the article were not sufficient. When overlapping data exists, only the latest study with the largest sample was selected for this meta-analysis. The last search date was July 1, 2013.

### Inclusion and Exclusion Criteria

Studies included in the present meta-analysis must meet the inclusion criteria as follows: observational studies concerning the association between *NOD2* gene polymorphisms (rs2066842 C/T, rs2066844 C/T, rs2066845 C/G and rs2066847 insC) and cancer risks; studies published in English; studies with sufficient raw data for estimating odds ratios (OR) and their 95% confidence interval (CI); the control group of the studies should be in accordance with the Hardy Weinberg Equilibrium (HWE). The main reasons for exclusion were reviews or meta-analysis; animal experiments; not relevant to specific polymorphisms; duplicate publications; no raw data after contacting the author; studies not in English.

### Data Extraction

Two authors (Jingwei Liu and Caiyun He) extracted the data from the included studies independently. The following information was extracted from each study: first author, year of publication, ethnicity of the population, numbers of cases and controls, detection methods of *NOD2* polymorphism and the source of the control group. The conflicts were resolved after discussion and consensus was finally reached on all of the extracted data.

### Statistical Analysis

The statistical analysis was performed by Stata software (Version 11.0; StataCorp, College Station, TX). ORs and their 95% CI were used to assess the strength of association between *NOD2* gene polymorphisms and cancer risks. P value <0.05 was considered as statistically significant. Heterogeneity was measured by using Q statistic (P<0.10 indicates significant heterogeneity between studies) and I-squared (I^2^) value [9]. A fixed-effects model using Mantel-Haenszel method [10] was performed to calculate the pooled ORs when heterogeneity between studies was not significant. Otherwise, a random-effects model using DerSimonian and Laird method [11] was applied. Sensitivity analysis was performed to explore heterogeneity when significant heterogeneity was indicated. Subgroup analyses were performed to explore the effects of cancer type and source of controls. Additionally, publication bias were evaluated qualitatively by performing funnel plots and assessed quantitatively by Begg’s test [12] and Egger’s test [13], respectively. P value<0.05 for Begg’s and Egger’s tests indicates significant publication bias.

## Results

### Characteristics of the Included Studies

This meta-analysis was organized according to the PRISMA statement (Checklist S1). Totally 93 literatures were indentified through electronic databases after duplicates removal. After reviewing the titles and abstracts of the potential available articles, 57 records were excluded mainly because of no relevance, in vitro or animal experiments, reviews or meta-analysis. The left 36 full-text articles were further assessed for eligibility. Finally, 30 full-text articles with eligibility were included in this meta-analysis [8], [14]–[42]. The flow chart of article selection was presented in Figure S1.

The main characteristics of the studies included in this meta-analysis were summarized in Table 1. All the included studies were case-control designed published in English. The populations of the studies were all Caucasians. Four studies consisting of 368 cases and 567 controls investigated the association of *NOD2* rs2066842 C/T (P268S) polymorphism and cancer risk; 16 studies including 4507 cases and 4780 controls studied the association of *NOD2* rs2066844 C/T (R702W) polymorphism and cancer risk; 14 articles including 4185 cases and 4474 controls investigated the association of *NOD2* rs2066845 C/G (G908R) polymorphism and cancer risk; for *NOD2* rs2066847/rs5743293 (3020insC) polymorphism, 25 studies consisting of 23167 cases and 28601 controls were included. The types of cancers studied in relation to *NOD2* polymorphisms included gastric cancer and MALT lymphoma, colorectal cancer (CRC), melanoma, endometrial cancer, pancreatic cancer, breast cancer, non-Hodgkin lymphoma, laryngeal cancer, lymphocytic leukaemia and ovarian cancer. Data concerning different cancers were treated as separate studies in the subgroup analysis.

**Table 1:** Characteristics of eligible studies in this meta-analysis.

Author	Year	Ethnicity	Cancer type	Controls source	Case	Control	Genotyping method
rs2066842 C/T (P268S)							
Roberts, R. L.	2006	New Zealander	Colorectal cancer	PB	133	201	ARMS
Wex, T.	2008	German	Gastric cancer	PB	167	153	PCR-RFLP
Szeliga, J.	2008	Polish	Rectal cancer	HB	51	100	PCR-RFLP
Hnatyszyn, A.	2010	Polish	Gastric cancer	HB	17	113	Pyrosequencing
rs2066844 C/T (R702W)							
Debniak, T.	2005	Polish	Melanoma	HB	470	649	Allele-specific PCR
Papaconstantinou, I.	2005	Greek	Colorectal cancer	N.A.	104	100	Allele-specific PCR
Rosenstiel, P.	2006	German	Gastric MALT lymphoma	HB	83	308	Taqman
Roberts, R. L.	2006	New Zealander	Colorectal cancer	PB	133	201	ARMS
Lakatos, P. L.	2007	Hungarian	Colorectal cancer	N.A.	194	200	dHPLC
Vogel, U.	2007	Danish	Colorectal cancer	PB	355	753	CE-SSCP
Tuupanen, S.	2007	Finnish	Colorectal cancer	PB	953	508	ARMS
Szeliga, J.	2008	Polish	Rectal cancer	HB	51	100	PCR-RFLP
Suchy, J.	2008	Polish	Colorectal cancer	HB	350	350	PCR-RFLP
Ture-Ozdemir, F.	2008	Greek	Gastric MALT lymphoma	HB	56	51	PCR-RFLP
Wex, T.	2008	German	Gastric cancer	PB	159	150	PCR-RFLP
Mockelmann, N.	2009	German	Colorectal cancer	PB	1044	724	SNPlex
Angeletti, S.	2009	Italian	Gastric cancer	PB	170	156	PCR-ARMS
Freire, P.	2010	Portuguese	Colorectal cancer	PB	112	152	Real-time PCR
Rigoli, L.	2010	Italian	Gastric cancer	PB	60	87	PCR-RFLP
Ashton, K. A.	2010	Austrilian	Endometrial cancer	PB	213	291	Real-time PCR
rs2066845 C/G (G908R)							
Papaconstantinou, I.	2005	Greek	Colorectal cancer	N.A.	104	100	PCR-RFLP
Debniak, T.	2005	Polish	Melanoma	HB	470	649	Allele-specific PCR
Rosenstiel, P.	2006	German	Gastric MALT lymphoma	HB	83	308	Taqman
Roberts, R. L.	2006	New Zealander	Colorectal cancer	PB	133	201	ARMS
Tuupanen, S.	2007	Finnish	Colorectal cancer	PB	960	508	ARMS
Lakatos, P. L.	2007	Hungarian	Colorectal cancer	N.A.	194	200	dHPLC
Vogel, U.	2007	Danish	Colorectal cancer	PB	355	753	CE-SSCP
Ture-Ozdemir, F.	2008	Greek	Gastric MALT lymphoma	HB	56	51	PCR-RFLP
Szeliga, J.	2008	Polish	Rectal cancer	HB	51	100	PCR-RFLP
Suchy, J.	2008	Polish	Colorectal cancer	HB	350	350	PCR-RFLP
Mockelmann, N.	2009	German	Colorectal cancer	PB	1044	724	SNPlex
Freire, P.	2010	Portuguese	Colorectal cancer	PB	112	152	Real-time PCR
Rigoli, L.	2010	Italian	Gastric cancer	PB	60	87	PCR-RFLP
Ashton, K. A.	2010	Austrilian	Endometrial cancer	PB	213	291	Real-time PCR
rs2066847/rs5743293 (3020insC)							
Nej, K.	2004	Polish	Pancreatic cancer	HB	127	300	Allele-specific PCR
Kurzawski, G.	2004	Polish	Colorectal cancer	HB	556	300	Allele-specific PCR
Alhopuro, P.	2004	Finnish	Colorectal cancer	PB	926	348	Allele-specific PCR
Huzarski, T.	2005	Polish	Breast cancer	HB	462	1910	Allele-specific PCR
Debniak, T.	2005	Polish	Melanoma	HB	470	649	Allele-specific PCR
Lubinski, J.	2005	Polish	Mixed	HB	2850	1910	Allele-specific PCR
Papaconstantinou, I.	2005	Greek	Colorectal cancer	N.A.	104	100	Allele-specific PCR
Rothman, N.	2006	Caucasian	Non-Hodgkin lymphoma	PB/HB	3069	3497	Mixed
Forrest, M. S.	2006	British, American	Non-Hodgkin lymphoma	PB	899	1433	Taqman
Roberts, R. L.	2006	New Zealander	Colorectal cancer	PB	133	201	ARMS
Jaworowska, E.	2006	Polish	Laryngeal cancer	HB	347	4102	Allele-specific PCR
Irmejs, A.	2006	Latvian	Mixed	PB	420	974	Allele-specific PCR
Lener, M. R.	2006	Polish	Mixed	HB	4496	2068	Allele-specific PCR
Lakatos, P. L.	2007	Hungarian	Colorectal cancer	N.A.	194	200	dHPLC
Vogel, U.	2007	Danish	Colorectal cancer	PB	355	753	CE-SSCP
Ennas, M. G.	2008	Italian	Lymphocytic leukaemia	PB	39	109	Taqman
Magnowski, P.	2008	Polish	Ovarian cancer	HB	257	1910	Allele-specific PCR
Szeliga, J.	2008	Polish	Rectal cancer	HB	51	100	ASA
Suchy, J.	2008	Polish	Colorectal cancer	HB	607	607	PCR-RFLP
Wex, T.	2008	German	Gastric cancer	PB	47	48	PCR-RFLP
Ture-Ozdemir, F.	2008	Greek	Gastric MALT lymphoma	HB	56	51	PCR-RFLP
Angeletti, S.	2009	Italian	Gastric cancer	PB	170	156	Multiplex PCR
Rigoli, L.	2010	Italian	Gastric cancer	PB	60	87	PCR-RFLP
Skibola, C. F.	2010	Mixed	Non-Hodgkin lymphoma	PB/HB	6360	6636	Mixed
Freire, P.	2010	Portuguese	Colorectal cancer	PB	112	152	Real-time PCR

Abbreviations: PB: population-based; HB: hospital-based.

### Associations of *NOD2* Polymorphisms with Cancer Risks

**Figure 1:**
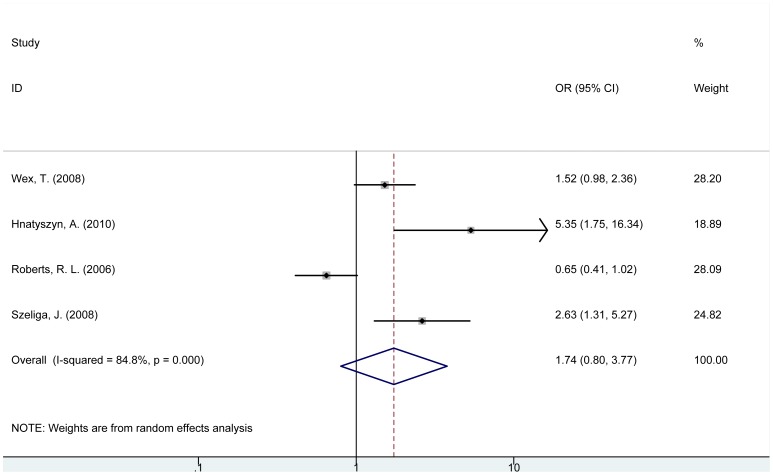
Forest plot for the association between *NOD2* rs2066842 polymorphism and cancer risk (TT+CT vs. CC).

For *NOD2* rs2066842 C/T (P268S) polymorphism, carriers of TT or CT genotype were not significantly associated with cancer risk compared with wild-type CC genotype (TT vs. CC: OR = 2.48, 95% CI = 0.85–7.25, P = 0.097; CT vs. CC: OR = 1.32, 95% CI = 0.54–3.25, P = 0.543, Table 2). Similarly, no significant relation was found in recessive effect model of (TT+CT) genotype comparing with CC genotype (OR = 1.74, 95% CI = 0.80–3.77, P = 0.163, Figure 1) or in allele analysis of T allele comparing with C allele (OR = 1.54, 95% CI = 0.74–3.21, P = 0.247) (Table 2). Results from the subgroup analysis of *NOD2* rs2066842 polymorphism were presented in Table S1.

**Table 2:** Meta-analysis results of the association between *NOD2* polymorphisms and cancer risks.

	Data set number	OR(95% CI)	P value	Model	P_het_	I^2^(%)
rs2066842 C/T (P268S)						
TT vs. CC	3	2.48(0.85–7.25)	0.097	R	0.041	68.8%
CT vs. CC	3	1.32(0.54–3.25)	0.543	R	0.002	83.9%
(TT+CT) vs. CC	4	1.74(0.80–3.77)	0.163	R	<0.001	84.8%
T allele vs. C allele	3	1.54(0.74–3.21)	0.247	R	<0.001	87.6%
rs2066844 C/T (R702W)						
TT vs. CC	7	**3.77(1.30–10.93)**	**0.015**	F	0.970	0.0%
CT vs. CC	13	**1.34(1.01–1.76)**	**0.040**	R	0.033	46.5%
(TT+CT) vs. CC	16	**1.32(1.01–1.72)**	**0.041**	R	0.012	49.9%
T allele vs. C allele	13	**1.43(1.09–1.88)**	**0.010**	R	0.024	48.8%
rs2066845 C/G (G908R)						
CG vs. GG	11	**1.39(1.03–1.87)**	**0.030**	F	0.186	27.1%
(CC+CG) vs. GG	14	**1.32(1.01–1.72)**	**0.040**	F	0.216	21.9%
C allele vs. G allele	11	**1.40(1.05–1.88)**	**0.024**	F	0.235	21.8%
rs2066847/rs5743293 (3020insC)						
+/+ vs. −/−	4	**3.42(1.59–7.40)**	**0.002**	F	0.514	0.0%
+/− vs. −/−	14	**1.35(1.06–1.72)**	**0.016**	R	0.010	54.0%
(+/+ and +/−) vs. −/−	25	**1.23(1.10–1.38)**	**<0.001**	R	0.092	28.6%
+ vs. −	13	**1.40(1.11–1.76)**	**0.004**	R	0.014	52.4%

R: random effect model; F: fixed effect model.

**Figure 2:**
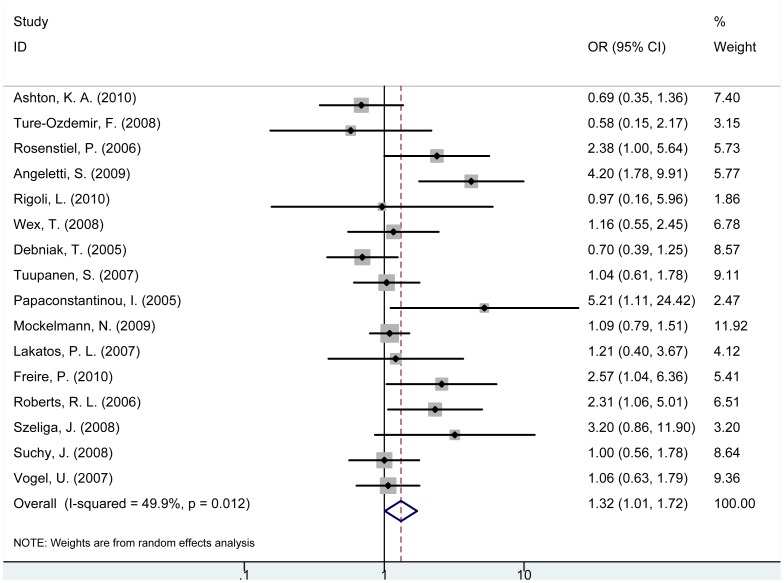
Forest plot for the association between *NOD2* rs2066844 polymorphism and cancer risk (TT+CT vs. CC).

For *NOD2* rs2066844 C/T (R702W) polymorphism, individuals with TT or CT genotype were associated with increased risk of cancer compared with CC carriers, respectively (TT vs. CC: OR = 3.77, 95% CI = 1.30–10.93, P = 0.015; CT vs. CC: OR = 1.34, 95% CI = 1.01–1.76, P = 0.040, Table 2). (TT+CT) genotype was associated with increased risk of cancer compared with wild-type CC genotype (OR = 1.32, 95% CI = 1.01–1.72, P = 0.041, Figure 2). In the subgroup analysis of cancer type, (TT+CT) genotype was associated with significantly increased risk of CRC (OR = 1.26, 95% CI = 1.03–1.53, P = 0.027) but no significant association was observed for gastric tumors (Table S2). In addition, T allele of *NOD2* rs2066844 C/T polymorphism was associated with significantly increased risk of cancer compared with C allele (Table 2).

**Figure 3:**
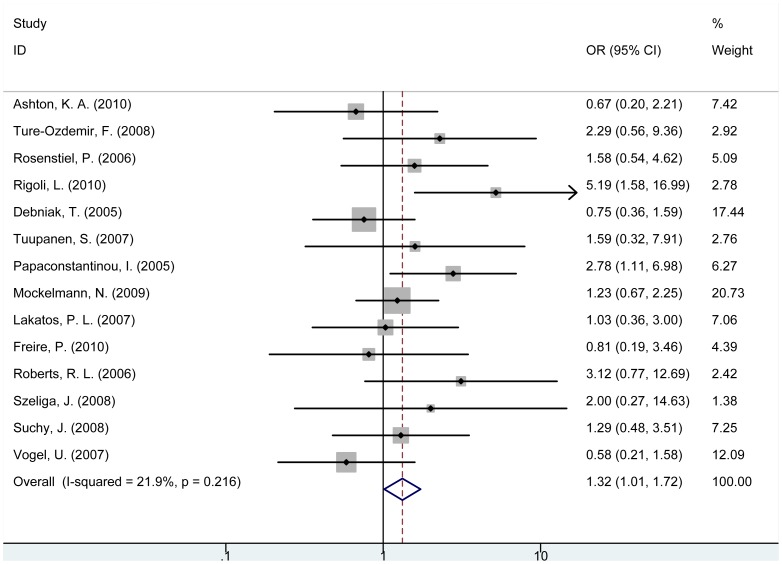
Forest plot for the association between *NOD2* rs2066845 polymorphism and cancer risk (CC+CG vs. GG).

For *NOD2* rs2066845 C/G (G908R) polymorphism, CG genotype carriers were observed to be significantly associated with increased risk of cancer compared with GG carriers (OR = 1.39, 95% CI = 1.03–1.87, P = 0.030, Table 2). Individuals with (CC+CG) genotype were significantly associated with increased risk of cancer in the overall analysis (OR = 1.32, 95% CI = 1.01–1.72, P = 0.040, Figure 3) and in gastric tumor subgroup (OR = 2.70, 95% CI = 1.39–5.25, P = 0.003, Table S3), but no significant association was observed in CRC subgroup. Additionally, C allele was associated with increased cancer risk compared with G allele.

**Figure 4:**
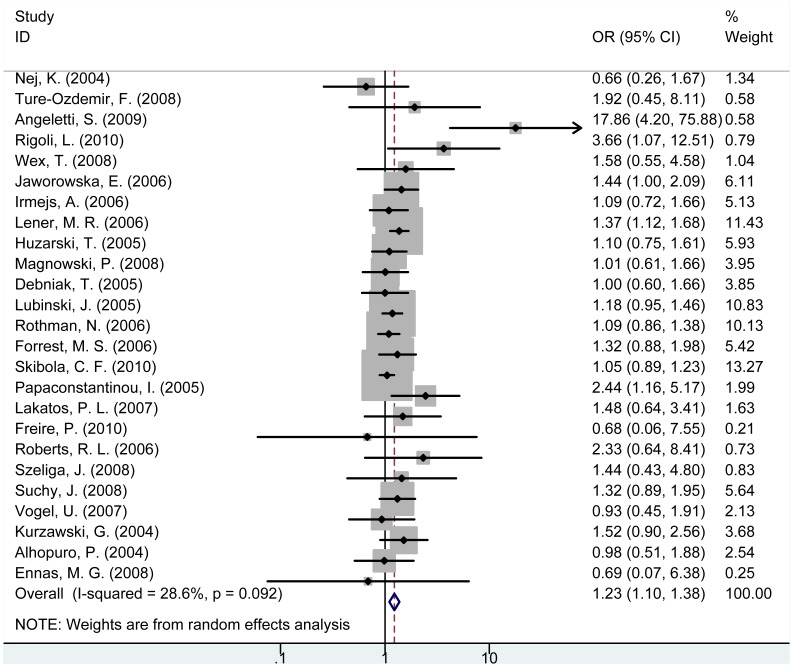
Forest plot for the association between *NOD2* rs2066847 polymorphism and cancer risk ((+/+ and +/−) vs. −/−).

For *NOD2* rs2066847/rs5743293 (3020insC) polymorphism, carriers of insC/insC or insC/− genotype were associated with increased cancer risk compared with wild-type −/− carriers, respectively (insC/insC vs. −/−: OR = 3.42, 95% CI = 1.59–7.40, P = 0.002; insC/− vs. −/−: OR = 1.35, 95% CI = 1.06–1.72, P = 0.016). Individuals with (insC/insC+insC/−) genotype were significantly associated with increased risk of cancer compared with −/− carriers (OR = 1.23, 95% CI = 1.10–1.38, P<0.001, Figure 4). In the subgroup analysis of cancer type, (insC/insC+insC/−) genotype was significantly associated with increased risk of colorectal cancer, gastric cancer and MALT lymphoma, breast cancer, lung cancer, laryngeal cancer but not with urogenital cancer, pancreatic cancer, melanoma or non-Hodgkin lymphoma (Table S4). In the subgroup analysis of control source, (insC/insC+insC/−) genotype was significantly associated with increased cancer risk in hospital-based subgroup (OR = 1.25, 95% CI = 1.12–1.40, P<0.001) but no significant association was observed in population-based subgroup (Table S4).

### Heterogeneity Test, Sensitivity Analysis, and Publication Bias

For most comparisons for *NOD2* gene rs2066844 C/T, rs2066845 C/G and rs2066847 insC polymorphisms, no obvious heterogeneity was observed (I^2^<50%). The exclusion of each single study did not significantly change the overall outcome, suggesting that the results of the meta-analysis were robust. However, in most comparisons of rs2066842 C/T polymorphism, significant heterogeneity was observed, which could not be fully explained by study design or subgroup analysis. The heterogeneity might result from the limited number of studies included. Besides, meta-regression was not performed to explore the source of the heterogeneity due to the limited study number.

The Begg’s test and Egger’s test were performed to quantitatively evaluate the publication bias of the studies. The detailed results for publication bias test were summarized in Table 3. No significant publication bias was observed in this meta-analysis except two comparisons in *NOD2* rs2066847 insC polymorphism. In addition, funnel plots that qualitatively evaluated the publication bias of association between *NOD2* rs2066847 insC polymorphism and cancer risk was presented in Figure S2.

**Table 3:** Publication bias.

	Compared genotype	Begg's test		Egger's test	
		z value	P value	t value	P value
rs2066842 C/T (P268S)	TT vs. CC	0.52	0.602	1.35	0.405
	CT vs. CC	0.52	0.602	0.87	0.546
	(TT+CT) vs. CC	1.36	0.174	1.70	0.231
	T allele vs. C allele	0.52	0.602	1.04	0.488
rs2066844 C/T (R702W)	TT vs. CC	-0.75	0.453	0.00	0.997
	CT vs. CC	1.83	0.067	1.96	0.076
	(TT+CT) vs. CC	1.53	0.126	1.79	0.095
	T allele vs. C allele	1.95	0.051	2.00	0.071
rs2066845 C/G (G908R)	CG vs. GG	0.39	0.697	0.45	0.666
	(CC+CG) vs. GG	0.93	0.352	1.05	0.313
	C allele vs. G allele	0.08	0.938	0.26	0.800
rs2066847/rs5743293 (3020insC)	+/+ vs. −/−	0.68	0.497	1.28	0.329
	+/− vs. −/−	1.46	0.143	2.53	**0.028**
	(+/+ and +/−) vs. −/−	1.03	0.304	1.88	0.073
	+ vs. −	1.10	0.272	2.42	**0.034**

## Discussion

Results from previous individual studies investigating the associations between *NOD2* polymorphisms and cancer risk were inconclusive. To our knowledge, this was the first comprehensive meta-analysis concerning the effect of *NOD2* rs2066842 C/T, rs2066844 C/T, rs2066845 C/G and rs2066847 insC polymorphisms on risks of overall cancer and specific cancer subtypes. By analyzing the data extracted from 30 full-text publications, we revealed that *NOD2* rs2066844 C/T, rs2066845 C/G and rs2066847 (3020insC) polymorphisms might be associated with increased cancer risk especially for gastrointestinal cancer but no significant association was observed between *NOD2* rs2066842 C/T polymorphism and cancer risk.

*NOD2* gene comprises 12 exons and encodes a protein consisting of 1040 amino acids. NOD2 recognized microbial pathogens located in the cytoplasm through the specific detection of conserved muramyl dipeptides and induced nuclear factor kappa B (NF-κB) activation via the RIP2/IKK pathway [43]. The NF-κB pathway acts to enhance the expression of proinflammatory molecules, thereby stimulating both adaptive and innate immune responses. In addition, NOD2 was implicated in programmed cell death and was known to be key regulator of chronic inflammatory conditions [44]. Recent attention has been given to the role of *NOD2* polymorphisms in carcinogenesis. Of which, four polymorphisms (rs2066842 C/T, rs2066844 C/T, rs2066845 C/G and rs2066847 insC) were of great interest. However, results of the individual studies came up with inconsistent conclusions.

In this meta-analysis, *NOD2* rs2066842 polymorphism was not observed to be associated with cancer risk in all comparisons. Only four studies were analyzed in the pooled estimates and obvious heterogeneities were detected which could not be explained by subgroup analysis or solved by meta-regression. Therefore, further large-scale studies were required to validate the results. With respect to *NOD2* rs2066844, rs2066845 and rs2066847 polymorphisms, the dominant effect models of the three polymorphisms all indicated significantly increased cancer risk (OR for rs2066844 = 1.32; OR for rs2066845 = 1.32; OR for rs2066847 = 1.23) (Table 2). The allele analysis found consistently increased cancer risk (OR for rs2066844 = 1.43; OR for rs2066845 = 1.40; OR for rs2066847 = 1.40) (Table 2).

Different cancer has its distinct mechanisms of initiation and progression, in which polymorphisms of key genes play a critical role. The present meta-analysis unraveled that *NOD2* polymorphisms were observed of different associations with cancer in various cancer subgroups. The (TT+CT) genotype of rs2066844 conferred increased risk to CRC but not gastric cancer and MALT lymphoma; individuals with (CC+CG) genotype of rs2066845 were associated with higher risk of gastric cancer and MALT lymphoma rather than CRC; (insC/insC+insC/−) genotype carriers of rs2066847 were associated with increased risk of colorectal cancer, gastric cancer and MALT lymphoma, breast cancer, lung cancer, laryngeal cancer but not with urogenital cancer, pancreatic cancer, melanoma or non-Hodgkin lymphoma. Besides, the source of controls adopted would influence the associations of polymorphisms with cancer risks. In the present study, (insC/insC+insC/−) genotype carriers of rs2066847 were associated with increased cancer risk in subgroup of hospital-based but not in population-based. The above-mentioned results of subgroup analysis uncovered underlying information and deserved future concerns.

Inflammation may lead to carcinogenesis by stimulating continuous cell proliferation, inducing DNA damage, and arousing angiogenesis [45]. Mutations of *NOD2* gene have been linked to a number of chronic inflammatory diseases including Crohn’s disease, atopic dermatitis and so on [46]. As NOD2 is implicated in microbial recognition and inflammatory reactions, the association of *NOD2* polymorphisms with cancer risk might due to the alteration of the ability of inducing immune response to bacteria which consequently results in the development of persistent bacterial infection or enhanced production of proinflammatory mediators. The three polymorphisms (rs2066844, rs2066845 and rs2066847) which were observed to be associated with increased risk of cancer in this meta-analysis were all located at the leucine-rich region (LRR) of NOD2 protein. The amino acid substitutions caused by these polymorphisms would alter protein function or splicing, thus influencing the role of NOD2 in the regulation of apoptosis and chronic inflammation and finally leading to cancer. As for functional studies, 3020insC variant, leading to a substitution in the 10th LRR followed by a premature stop codon, has been proved to be less active in the response to bacterial lipopolysaccharides, which might produce an increased inflammatory response [47]. Mice deficient in NOD1, NOD2, or RIPK2 exhibited increased susceptibility to bacteria, which arises from a decreased ability to recruit neutrophils and less production in proinflammatory and antimicrobial molecules [17], [48]. Although the above-mentioned studies could, at least in part, explain the observed relation of *NOD2* polymorphisms with cancer risk, future investigations concerning the specific mechanism of *NOD2* polymorphisms in carcinogenesis are still required.

Several limitations should be acknowledged in this meta-analysis when interpreting the results. First, the sample size was not sufficiently large for the pooled analysis of *NOD2* rs2066842 C/T polymorphism and some subgroup analyses for *NOD2* rs2066844 C/T, rs2066845 C/G and rs2066847 insC polymorphisms. Second, all the included studies in the current meta-analysis were published in English, therefore publication bias might exist although the statistical test did not indicate it. Third, the ethnicities of all the available studies were Caucasian populations, which inevitably limited the generalizability of our conclusion on other populations. Fourth, obvious heterogeneities were observed in a few comparisons, which would limit the accuracy of certain associations. Finally, as other important data of environment factors such as smoking or drinking were not available for individual studies, we could not obtained results with adjustments by other co-variables.

## Conclusion

To be concluded, this meta-analysis suggested that *NOD2* rs2066844 C/T, rs2066845 C/G and rs2066847 (3020insC) polymorphisms might be associated with increased cancer risk especially for gastrointestinal cancer. No significant association was observed between *NOD2* rs2066842 C/T polymorphism and cancer risk. Further large-scale and well-designed studies concerning different ethnicities are still needed to confirm the results of our meta-analysis.

## Supporting Information

Figure S1 **The flowchart of literature inclusion and exclusion.**
(TIF)

Figure S2 **Funnel plot for studies of association between NOD2 rs2066847 polymorphism and cancer risk ((+/+ and +/−) vs. −/−).**
(TIF)

Table S1 **Subgroup analysis of association between** ***NOD2*** **rs2066842 polymorphism and cancer risk.**
(DOC)

Table S2 **Subgroup analysis of association between** ***NOD2*** **rs2066844 polymorphism and cancer risk.**
(DOC)

Table S3 **Subgroup analysis of association between** ***NOD2*** **rs2066845 polymorphism and cancer risk.**
(DOC)

Table S4 **Subgroup analysis of association between** ***NOD2*** **rs2066847 polymorphism and cancer risk.**
(DOC)

Checklist S1 **PRISMA checklist.**
(DOC)
