# Supplementary figures and images for: NOD2 Polymorphisms Associated with Cancer Risk: A Meta-Analysis
Source: PLoS One. 2014 Feb 20;9(2):e89340. doi: 10.1371/journal.pone.0089340 (PMC3930717; doi:10.1371/journal.pone.0089340)

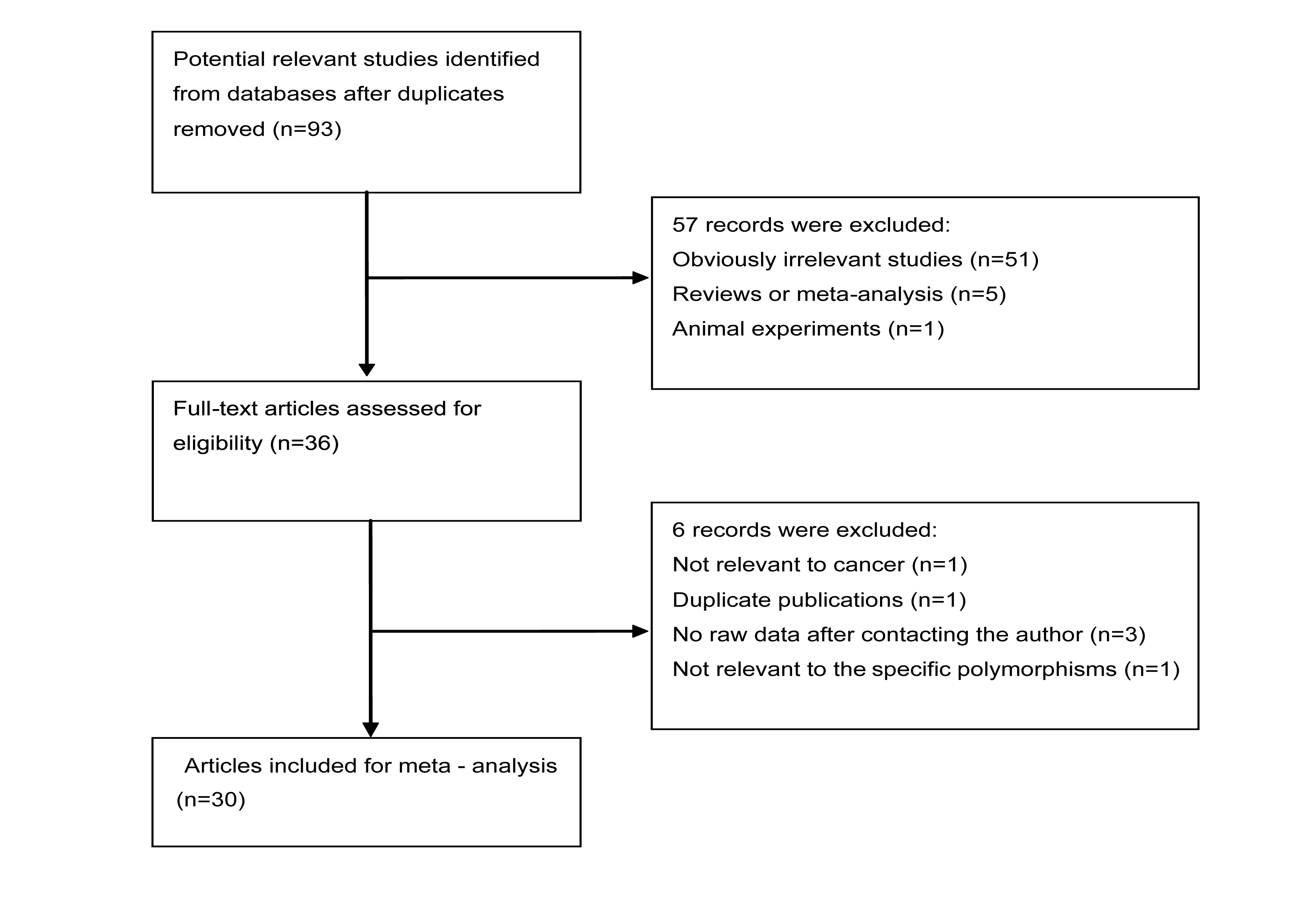

Supplement: Figure S1 — The flowchart of literature inclusion and exclusion. (TIF) [file pone.0089340.s001.tif]

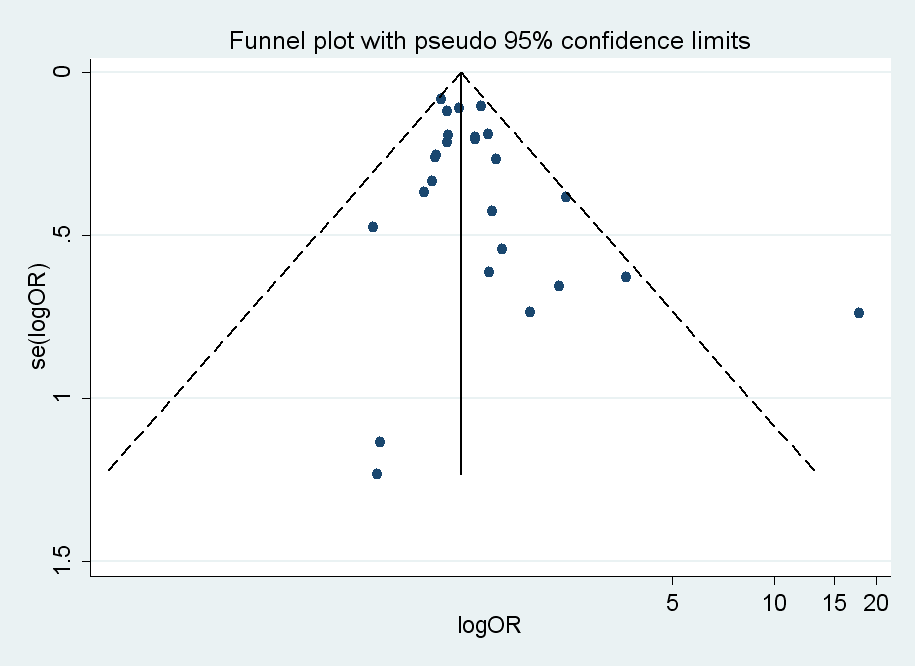

Supplement: Figure S2 — Funnel plot for studies of association between NOD2 rs2066847 polymorphism and cancer risk ((+/+ and +/−) vs. −/−). (TIF) [file pone.0089340.s002.tif]
