# Supplementary material for: NOD2 Polymorphisms Associated with Cancer Risk: A Meta-Analysis
Source: PLoS One. 2014 Feb 20;9(2):e89340. doi: 10.1371/journal.pone.0089340 (PMC3930717; doi:10.1371/journal.pone.0089340)
Supplement: Table S1 — Subgroup analysis of association between NOD2 rs2066842 polymorphism and cancer risk. (DOC) [file pone.0089340.s003.doc]

Table S1. Subgroup analysis of association between *NOD2* rs2066842 polymorphism and cancer risk

| Subgroup | Compared genotype | Study number | OR(95%CI) | P value | Model | P_het_ | I^2^(%) |
| --- | --- | --- | --- | --- | --- | --- | --- |
| Gastric cancer | TT vs. CC | 2 | 4.20(0.98-18.00) | 0.053 | R | 0.092 | 64.9% |
|  | CT vs. CC | 2 | 2.13(0.73-6.20) | 0.165 | R | 0.085 | 66.2% |
|  | (TT+CT) vs. CC | 2 | 2.56(0.76-8.62) | 0.130 | R | 0.040 | 76.3% |
|  | T allele vs. C allele | 2 | 2.27(0.84-6.13) | 0.104 | R | 0.016 | 82.9% |
| Colorectal cancer | TT vs. CC | 1 | 1.06(0.44-2.56) | 0.904 | / | / | / |
|  | CT vs. CC | 1 | **0.59(0.36-0.95)** | **0.030** | / | / | / |
|  | (TT+CT) vs. CC | 2 | 1.27(0.32-5.00) | 0.733 | R | 0.001 | 90.8% |
|  | T allele vs. C allele | 1 | 0.78(0.54-1.13) | 0.189 | / | / | / |
| PB | TT vs. CC | 2 | 1.61(0.89-2.91) | 0.116 | F | 0.211 | 36.1% |
|  | CT vs. CC | 2 | 0.91(0.39-2.12) | 0.823 | R | 0.011 | 84.6% |
|  | (TT+CT) vs. CC | 2 | 0.99(0.43-2.29) | 0.987 | R | 0.008 | 85.7% |
|  | T allele vs. C allele | 2 | 1.07(0.58-1.96) | 0.829 | R | 0.015 | 82.9% |
| HB | TT vs. CC | 1 | **10.40(2.20-49.25)** | **0.003** | / | / | / |
|  | CT vs. CC | 1 | **4.30(1.30-14.23)** | **0.017** | / | / | / |
|  | (TT+CT) vs. CC | 2 | **3.22(1.79-5.79)** | **<0.001** | F | 0.289 | 11.0% |
|  | T allele vs. C allele | 1 | **4.01(1.89-8.52)** | **<0.001** | / | / | / |
