# Supplementary material for: NOD2 Polymorphisms Associated with Cancer Risk: A Meta-Analysis
Source: PLoS One. 2014 Feb 20;9(2):e89340. doi: 10.1371/journal.pone.0089340 (PMC3930717; doi:10.1371/journal.pone.0089340)
Supplement: Table S2 — Subgroup analysis of association between NOD2 rs2066844 polymorphism and cancer risk. (DOC) [file pone.0089340.s004.doc]

Table S2. Subgroup analysis of association between *NOD2* rs2066844 polymorphism and cancer risk

| Subgroup | Compared genotype | Study number | OR(95%CI) | P value | Model | P_het_ | I^2^(%) |
| --- | --- | --- | --- | --- | --- | --- | --- |
| Gastric tumor | TT vs. CC | 3 | 4.18(0.80-21.71) | 0.089 | F | 0.683 | 0.0% |
|  | CT vs. CC | 4 | 1.86(0.85-4.07) | 0.281 | R | 0.059 | 59.8% |
|  | (TT+CT) vs. CC | 5 | 1.65(0.84-3.24) | 0.148 | R | 0.069 | 54.1% |
|  | T allele vs. C allele | 4 | **2.16(1.41-3.32)** | **<0.001** | F | 0.238 | 29.0% |
| Colorectal cancer | TT vs. CC | 4 | 3.52(0.88-14.12) | 0.076 | F | 0.900 | 0.0% |
|  | CT vs. CC | 8 | 1.19(0.97-1.47) | 0.090 | F | 0.268 | 20.4% |
|  | (TT+CT) vs. CC | 9 | **1.26(1.03-1.53)** | **0.027** | F | 0.144 | 34.3% |
|  | T allele vs. C allele | 8 | **1.25(1.03-1.53)** | **0.025** | F | 0.129 | 37.7% |
| PB | TT vs. CC | 6 | **4.18(1.31-13.34)** | **0.016** | F | 0.969 | 0.0% |
|  | CT vs. CC | 9 | 1.26(0.92-1.73) | 0.153 | R | 0.047 | 49.0% |
|  | (TT+CT) vs. CC | 9 | 1.34(0.97-1.85) | 0.077 | R | 0.030 | 52.9% |
|  | T allele vs. C allele | 9 | **1.40(1.01-1.95)** | **0.041** | R | 0.020 | 55.9% |
| HB | TT vs. CC | 1 | 1.31(0.05-32.56) | 0.868 | / | / | / |
|  | CT vs. CC | 2 | 1.50(0.61-3.72) | 0.380 | R | 0.080 | 67.3% |
|  | (TT+CT) vs. CC | 5 | 1.17(0.67-2.04) | 0.592 | R | 0.064 | 55.0% |
|  | T allele vs. C allele | 2 | 1.24(0.77-1.99) | 0.369 | F | 0.136 | 55.0% |
