# Supplementary material for: NOD2 Polymorphisms Associated with Cancer Risk: A Meta-Analysis
Source: PLoS One. 2014 Feb 20;9(2):e89340. doi: 10.1371/journal.pone.0089340 (PMC3930717; doi:10.1371/journal.pone.0089340)
Supplement: Table S3 — Subgroup analysis of association between NOD2 rs2066845 polymorphism and cancer risk. (DOC) [file pone.0089340.s005.doc]

Table S3. Subgroup analysis of association between *NOD2* rs2066845 polymorphism and cancer risk

| Subgroup | Compared genotype | Study number | OR(95%CI) | P value | Model | P_het_ | I^2^(%) |
| --- | --- | --- | --- | --- | --- | --- | --- |
| Gastric tumor | CG vs. GG | 2 | **2.85(1.35-6.03)** | **0.006** | F | 0.144 | 53.3% |
|  | (CC+CG) vs. GG | 3 | **2.70(1.39-5.25)** | **0.003** | F | 0.337 | 8.0% |
|  | C allele vs. G allele | 2 | **2.74(1.31-5.73)** | **0.007** | F | 0.165 | 48.2% |
| Colorectal cancer | CG vs. GG | 8 | 1.29(0.92-1.81) | 0.139 | F | 0.397 | 4.3% |
|  | (CC+CG) vs. GG | 9 | 1.32(0.95-1.84) | 0.103 | F | 0.490 | 0.0% |
|  | C allele vs. G allele | 8 | 1.31(0.94-1.83) | 0.112 | F | 0.439 | 0.0% |
| PB | CG vs. GG | 7 | 1.31(0.74-2.34) | 0.352 | R | 0.091 | 45.0% |
|  | (CC+CG) vs. GG | 7 | 1.33(0.75-2.35) | 0.337 | R | 0.092 | 44.8% |
|  | C allele vs. G allele | 7 | 1.29(0.90-1.87) | 0.168 | F | 0.111 | 42.0% |
| HB | CG vs. GG | 2 | 1.41(0.68-2.95) | 0.358 | F | 0.168 | 47.4% |
|  | (CC+CG) vs. GG | 5 | 1.17(0.74-1.87) | 0.502 | F | 0.588 | 0.0% |
|  | C allele vs. G allele | 2 | 1.40(0.68-2.91) | 0.362 | F | 0.795 | 0.0% |
