# Supplementary material for: NOD2 Polymorphisms Associated with Cancer Risk: A Meta-Analysis
Source: PLoS One. 2014 Feb 20;9(2):e89340. doi: 10.1371/journal.pone.0089340 (PMC3930717; doi:10.1371/journal.pone.0089340)
Supplement: Table S4 — Subgroup analysis of association between NOD2 rs2066847 polymorphism and cancer risk. (DOC) [file pone.0089340.s006.doc]

Table S4. Subgroup analysis of association between *NOD2* rs2066847 polymorphism and cancer risk

| Subgroup | Compared genotype | Study number | OR(95%CI) | P value | Model | P_het_ | I^2^(%) |
| --- | --- | --- | --- | --- | --- | --- | --- |
| Colorectal cancer | +/- vs. -/- | 6 | **1.38(1.04-1.83)** | **0.025** | F | 0.467 | 0.0% |
|  | (+/+ and +/- ) vs. -/- | 11 | **1.38(1.14-1.66)** | **0.001** | F | 0.597 | 0.0% |
|  | + vs. - | 6 | **1.43(1.09-1.87)** | **0.011** | F | 0.572 | 0.0% |
| Gastric tumor | +/- vs. -/- | 3 | **4.37(1.15-16.63)** | **0.031** | R | 0.030 | 71.4% |
|  | (+/+ and +/- ) vs. -/- | 5 | **2.68(1.16-6.20)** | **0.021** | R | 0.013 | 68.2% |
|  | + vs. - | 3 | **4.04(1.10-14.83)** | **0.035** | R | 0.031 | 71.3% |
| Breast cancer | (+/+ and +/- ) vs. -/- | 4 | **1.27(1.09-1.48)** | **0.002** | F | 0.608 | 0.0% |
| Lung cancer | (+/+ and +/- ) vs. -/- | 2 | **1.41(1.06-1.89)** | **0.019** | F | 0.309 | 3.2% |
| Urogenital cancer | (+/+ and +/- ) vs. -/- | 4 | 1.06(0.65-1.73) | 0.802 | R | 0.014 | 71.9% |
| Pancreatic cancer | (+/+ and +/- ) vs. -/- | 2 | 0.64(0.34-1.19) | 0.161 | F | 0.938 | 0.0% |
| Laryngeal cancer | (+/+ and +/- ) vs. -/- | 2 | **1.45(1.08-1.93)** | **0.012** | F | 0.979 | 0.0% |
| Melanoma | (+/+ and +/- ) vs. -/- | 2 | 0.85(0.57-1.27) | 0.428 | F | 0.346 | 0.0% |
| Non-Hodgkin lymphoma | +/+ vs. -/- | 3 | **3.18(1.43-7.09)** | **0.005** | F | 0.378 | 0.0% |
|  | +/- vs. -/- | 3 | 1.05(0.92-1.19) | 0.461 | F | 0.860 | 0.0% |
|  | (+/+ and +/- ) vs. -/- | 3 | 1.08(0.95-1.23) | 0.218 | F | 0.573 | 0.0% |
|  | + vs. - | 3 | 1.11(0.98-1.26) | 0.087 | F | 0.277 | 22.2% |
| PB | +/- vs. -/- | 8 | 1.82(0.98-3.36) | 0.056 | F | 0.013 | 60.7% |
|  | (+/+ and +/- ) vs. -/- | 10 | 1.49(1.00-2.22) | 0.052 | R | 0.025 | 52.8% |
|  | + vs. - | 8 | **1.85(1.07-3.21)** | **0.028** | R | 0.034 | 53.8% |
| HB | +/- vs. -/- | 1 | 1.26(0.85-1.87) | 0.259 | / | / | / |
|  | (+/+ and +/- ) vs. -/- | 11 | **1.25(1.12-1.40)** | **<0.001** | F | 0.783 | 0.0% |
|  | + vs. - | 1 | 1.37(0.94-2.00) | 0.106 | / | / | / |
